# Gut–liver axis dysregulation and microbial dysbiosis in invasive liver abscess: a narrative review

**DOI:** 10.3389/fimmu.2025.1646893

**Published:** 2025-10-29

**Authors:** Xiaoshuai Bai, Zhen Wang, Kai Guo, Ping Zhou, Lei Shi

**Affiliations:** ^1^ Department of Emergency Internal Medicine, The Affiliated Hospital of Qingdao University, Qingdao, China; ^2^ Department of Oncology, Qingdao Central Hospital, University of Health and Rehabilitation Sciences (Qingdao Central Hospital), Qingdao, China

**Keywords:** gut–liver axis dysregulation, invasive liver abscess, microbial dysbiosis, hypervirulent *Klebsiella pneumoniae*, clinical pathology

## Abstract

Invasive liver abscess (ILA) represents a formidable clinical challenge, characterized by rapidly evolving hepatic lesions and systemic dissemination. The gut–liver axis, a vital conduit for immune and metabolic regulation, has emerged as a central driver of its pathogenesis. This narrative review draws on insights from select transcriptomic, proteomic, metabolomic, and microbiomic studies, revealing how chronic antibiotic use, unhealthy diets, and lingering pathological conditions disrupt intestinal barrier integrity and perturb bile acid and short-chain fatty acid metabolism. This dysregulated microenvironment facilitates bacterial translocation into the liver, triggering a robust inflammatory cascade and the upregulation of virulence factors involved in capsule synthesis and biofilm formation. Evidence suggests microbial dysbiosis contributes to hepatic immune dysregulation. These insights pave the way for novel ILA interventions. This review offers original insights by critically integrating evidence from transcriptomic, proteomic, metabolomic, and microbiomic studies with GRADE-evaluated clinical data, proposing a novel bacteria–inflammation–virulence feedback loop and precision therapeutic frameworks that target the gut-liver axis, filling gaps in traditional ILA models and guiding future interventions.

## 1 Introduction

ILA is a rapidly progressive and pathologically complex infectious disease that has garnered increasing clinical attention (1, 2). In recent years, lifestyle changes, a rising incidence of metabolic disorders, and the widespread prevalence of risk factors such as diabetes, cirrhosis, advanced age, and immunocompromised conditions, have contributed to a higher prevalence of ILA (3). However, evidence from epidemiological studies is often retrospective and regionally biased (e.g., East Asian cohorts), warranting caution in global extrapolation (3); using GRADE criteria, this evidence is moderate-low due to potential confounding by comorbidities. This condition exhibits a high mortality rate and poses significant treatment challenges, given the limitations of conventional antibiotics and interventional therapies. Consequently, it is essential to explore pathogenic mechanisms, particularly the gut–liver axis and microbial dysbiosis, while critically evaluating the quality of supporting data.

The gut–liver axis, serving as a bidirectional conduit between the digestive system and the liver, plays a crucial role in maintaining immune homeostasis and metabolic balance (4). Extensive evidence indicates that disruptions in the gut microbiota, along with impaired intestinal barrier, enable the translocation of bacterial endotoxins and metabolic byproducts via the portal vein into the liver, thereby triggering local inflammatory responses and immune dysregulation (5–8). These citations primarily draw from mechanistic animal and *in vitro* models (5, 6), which provide high internal validity but limited human applicability; in contrast, clinical observations (7, 8) are observational and graded as low quality due to small sample sizes. This mechanism not only initiates localized infection but also significantly influences disease progression and recurrence. Accordingly, this review focuses on the critical role of gut–liver axis disruption and microbial imbalance in the pathophysiology of ILA, aiming to elucidate potential mechanisms and to offer novel insights for precision clinical interventions, such as early microbiome screening in at-risk patients to prevent translocation.

The objective of this review is to integrate recent international research on ILA, with a particular focus on the limitations of conventional antimicrobial and interventional therapies. In addition, we examine emerging treatment strategies, including probiotic supplementation, fecal microbiota transplantation, and multi-target combination therapies. By synthesizing and comparing how various therapeutic approaches modulate gut–liver axis function, restore intestinal microbial balance, and enhance immune regulation, our goal is to establish a comprehensive therapeutic framework. This framework not only provides a robust theoretical foundation for clinical practice but also offers practical guidance for devising individualized, multi-target treatment strategies, such as combining antibiotics with probiotics based on patient dysbiosis profiles. Literature published since 2000 was evaluated and screened to ensure the quality and representativeness of the included studies; however, we further assess evidence quality using GRADE and distinguish mechanistic from clinical data to highlight where firm conclusions can be drawn.

Consequently, this review comprehensively addresses the pathological mechanisms and conventional treatment strategies of ILA while highlighting the potential applications of novel interventions in modulating the gut–liver axis and restoring microbial homeostasis. The barrier of the gut–liver axis is critical for maintaining immune equilibrium and metabolic balance (9–11). However, various adverse factors can compromise the intestinal barrier, thereby allowing bacteria and their toxins to infiltrate the liver and trigger localized inflammatory responses (12, 13). Mechanistic studies (primarily *in vitro*) suggest direct barrier compromise (9–11), graded as moderate quality, while clinical implications (12, 13) remain speculative without large-scale RCTs. With this molecular framework of gut–liver axis dysregulation and microbial imbalance established, we now turn to the clinical pathology of invasive liver abscess and the limitations of traditional infection models.

## 2 Clinical pathology of invasive liver abscess

### 2.1 Limitations of traditional infection mechanisms

ILA has high mortality (14). Traditionally, it is attributed to two factors: pathogen hypervirulence and reduced host defenses such as diabetes, chronic liver disease, and immunodeficiency (15). However, an increasing number of recent clinical cases reveal that even patients with relatively normal immune function and no apparent hepatobiliary disease can manifest highly invasive pathology with multi-system dissemination (16–18). These observations suggest that conventional infection models, relying solely on pre-existing host conditions, are insufficient to explain ILA, particularly when severe systemic spread is observed in individuals without clear underlying disorders (19).

### 2.2 Unique pathogenic mechanisms of HvKP

Advances in molecular diagnostics and clinical studies highlight HvKP’s hypermucoviscosity (20), detected via the string test (viscous string > 5 mm). This phenotype reflects increased capsular polysaccharide synthesis and underpins its hypervirulence. Further molecular analyses have demonstrated that several key virulence genes are highly conserved among HvKP strains. For instance, plasmid-encoded genes such as *rmpA* (*prmpA*) and *rmpA2*, together with the chromosomal variant *rmpA* (*crmpA*), play pivotal roles in regulating capsule synthesis, thereby reinforcing the hypermucoviscosity phenotype and promoting immune evasion. In parallel, specific siderophore biosynthesis genes like *iucA* (responsible for aerobactin synthesis) and the putative transporter *peg-344* are intimately associated with the organism’s high pathogenicity. Additionally, epidemiologically relevant genes, including *terB* (conferring tellurite resistance), *iroB* (involved in salmochelin biosynthesis), and *irp2* (linked to yersiniabactin biosynthesis), are frequently detected in hypervirulent isolates. Based on these molecular markers, researchers have further delineated the capsular serotypes associated with these strains (such as K1, K2, K5, K20, K54, and K57), thereby providing a robust molecular framework for understanding their pathogenic mechanisms (21, 22). Clinically, these capsular overproductions and enhanced siderophore traits demand rapid molecular diagnostics to guide targeted antibiotic selection and improve patient outcomes.

### 2.3 Summary of clinical cases and pathological manifestations

In East Asia, particularly in Taiwan, infections caused by HvKP are increasingly observed in otherwise healthy individuals without evident hepatobiliary disease. These patients typically exhibit several defining characteristics (23–25): (1) Local Manifestations: Hepatic abscesses are often multi-focal, presenting as either localized or diffusely infiltrative lesions accompanied by pronounced acute inflammatory responses. (2) Systemic Dissemination: Beyond the primary hepatic lesions, patients frequently develop multi-system infections, including meningitis, endophthalmitis, empyema, septic pulmonary emboli, septic arthritis, osteomyelitis, necrotizing fasciitis, and bloodstream infections. These disseminated infections tend to progress rapidly and are associated with a poor prognosis. (3) Abnormal Clinical Indicators: Laboratory tests commonly reveal fever, leukocytosis, and impaired liver function, all of which signal a marked activation of the inflammatory response. These clinical patterns collectively point toward gut–liver axis disruption, a link we mechanistically explore in Section 3.

### 2.4 Summary of molecular detection and virulence genes

Molecular diagnostic studies of HvKP have demonstrated that the upregulation of multiple virulence genes is closely associated with its enhanced pathogenicity. The table below summarizes the key genes commonly detected in HvKP isolates and outlines their roles in the pathogen’s virulence mechanisms (**Table 1**).

**Table 1:** Gene function and pathogenic mechanism table.

Gene	Function	Role in pathogenic mechanisms	Additional notes
*rmpA*/*crmpA*	Regulator of Capsule Synthesis	Upregulates the production of capsular polysaccharides, thereby enhancing the hypermucoviscous phenotype and promoting immune evasion.	Detected on both plasmid (*prmpA*) and chromosome (*crmpA*).
*rmpA2*	Capsule Regulation	Functions similarly to *rmpA* by promoting capsule formation and thereby contributing to increased virulence.	Typically plasmid-encoded.
*iucA*	Siderophore Biosynthesis	Involved in the synthesis of aerobactin, a siderophore that facilitates iron acquisition essential for bacterial growth.	Closely associated with elevated virulence.
*peg-344*	Putative Transporter	Serves as a molecular marker for hypervirulence; Although its precise function remains under investigation, it is linked with aggressive infection.	Emerging as an important indicator of HvKP.
*terB*	Tellurite Resistance	Confers resistance to tellurite and may contribute to survival under environmental stress, indirectly supporting virulence. Confers resistance to tellurite and may support bacterial survival under environmental stress, indirectly enhancing virulence.	Frequently detected in hypervirulent strains.
*iroB*	Siderophore Biosynthesis	Plays a critical role in salmochelin biosynthesis, aiding in iron acquisition and promoting pathogenicity.	Acts as an epidemiological marker for virulence.
*irp2*	Siderophore Biosynthesis	Participates in yersiniabactin synthesis, which is crucial for iron uptake and overall strain virulence.	Commonly observed in HvKP isolates.

To aid readers who are not familiar with microbial genetics, a concise glossary of the most clinically relevant virulence genes is provided below.

To aid readers who are not familiar with microbial genetics, a concise glossary of the most clinically relevant virulence genes is provided below (**Table 2**). While molecular assays pinpoint key virulence genes, an integrated gut–liver axis perspective reveals how dysbiosis drives disease *in vivo*.

**Table 2:** Glossary of key virulence genes in *Klebsiella pneumoniae*.

Gene	Full name/Type	Main function	Role in virulence and clinical relevance
*rmpA*	Regulator of mucoid phenotype A (plasmid-encoded or chromosomal variant)	Activates transcription of capsule polysaccharide synthesis genes	Increases capsule thickness, enhances hypermucoviscosity, protects bacteria from host immunity, linked to invasive liver abscess
*rmpA2*	Regulator of mucoid phenotype A2 (usually plasmid-encoded)	Similar to *rmpA*, promotes capsule production	Strengthens capsule-mediated immune evasion, often coexists with *rmpA*, serves as a molecular marker for hypervirulent strains
*iucA*	Aerobactin synthetase gene, part of the *iucABCD* operon	Encodes enzyme for aerobactin (a siderophore) synthesis	Facilitates iron acquisition under host-limited conditions, significantly enhances bacterial growth and invasiveness

### 2.5 The role of the gut-liver axis and dysbiosis

Recent studies implicate gut–liver dysregulation in ILA pathogenesis. Traditional models focus on direct pathogen invasion and host immunodeficiency but overlook how microbiota imbalance and barrier breakdown enable bacterial translocation (26–28). However, these studies (26–28) are primarily mechanistic, relying on animal models with high internal validity but potential overestimation of translocation rates in humans; graded as moderate quality under GRADE due to lack of randomization.

Emerging research indicates that multiple factors collectively promote the onset of ILA: (1) Disruption of the Intestinal Barrier: Impairments in mucosal barrier, attributable to factors such as medication use, dietary changes, or other pathological conditions, permit highly pathogenic bacterial strains to enter the bloodstream, thereby seeding infections in the liver and other organs. (2) Microbial Dysbiosis: Alterations in the composition of the intestinal microbiota favor the predominance of harmful bacteria, notably HvKP, thereby increasing the risk of these pathogens translocating into systemic circulation. (3) Systemic Inflammatory Response: Both local and systemic inflammatory states further disturb homeostasis, compromising host immune defenses against highly virulent strains and exacerbating disease progression (29–32). These studies (29–32) include clinical case series (low GRADE quality due to small samples and biases) alongside *in vitro* data, highlighting a need to distinguish: mechanistic evidence firmly supports barrier roles, while clinical data speculatively links dysbiosis to dissemination in healthy hosts. This integrated perspective, combining direct pathogen invasion with host environmental alterations, not only provides novel molecular and immunological insights into the acute multi-system dissemination observed in patients without underlying conditions but also outlines promising avenues for future therapeutic strategies (33). For example, the integration of host immunomodulatory measures, restoration of intestinal barrier, and targeted interventions against pathogen-associated intracellular signaling pathways may represent key breakthroughs in reducing mortality and preventing systemic dissemination (34–37); practically, this suggests early probiotic use in high-risk groups, though RCTs are needed for validation.

Cumulative clinical evidence and molecular diagnostics consistently indicate that HvKP plays a predominant role in the pathogenesis of ILA (38). Although traditional models partially account for the roles of direct bacterial invasion and host immunodeficiency (39), they fall short of explaining the invasive, multisystem dissemination seen in otherwise healthy individuals. In contrast, the pronounced pathogenicity of hypermucoviscous strains, with their distinctive virulence gene expression profiles and heightened sensitivity to inflammatory signals, offers a fresh perspective for elucidating this complex pathology (40, 41). Furthermore, disruptions of the gut–liver axis, combined with dysbiosis and intestinal barrier damage, provide a robust framework for understanding the complex pathogenic mechanisms involved. To clarify evidence types, **Table 3** compares mechanistic and clinical studies in this context.

**Table 3:** Comparison of mechanistic vs. clinical studies on gut-liver axis in ILA.

Aspect	Mechanistic studies (*in vitro*/animal)	Clinical studies (human cohorts)	Implications
Barrier Disruption	Animal models show antibiotic-induced dysbiosis leading to translocation (26–28); high reproducibility but artificial.	Case series link diet/chronic disease to barrier failure (29–32); low GRADE, confounding by comorbidities.	Firm: Translocation mechanism; Speculative: Prevalence in healthy humans.
HvKP Role	*In vitro* gene upregulation by inflammation (40, 41); controlled but lacks *in vivo* complexity.	Retrospective cohorts show predominance in East Asia (38, 39); moderate GRADE, geographic bias.	Firm: Virulence in models; Speculative: Universal applicability without global RCTs.

## 3 Mechanism analysis

In recent years, mounting evidence has underscored the critical role of the gut–liver axis in maintaining hepatic immune and metabolic homeostasis (4). Disruption of the intestinal microbiota, coupled with compromised epithelial barrier, plays a pivotal role in the development of ILA (42). In this section, we aim to elucidate the underlying mechanisms by delineating the complex interrelationships among gut–liver axis disruption, abnormal gut microbiota, and the pathogenesis and progression of ILA. The discussion is organized around several principal regulatory pathways, including intestinal barrier disruption, the activation of inflammatory signaling cascades, and the bacteria–inflammation–virulence feedback loop.

### 3.1 Intestinal barrier disruption

#### 3.1.1 Normal gut microbial ecosystem, metabolic products, and barrier

The human gut contains ~10¹^4^ microbes, over 90% from Bacteroidetes and Firmicutes, alongside fungi, archaea, viruses, and protozoa (12, 43, 44). These communities maintain host health via metabolic and immune interactions. A healthy gut is equipped with multiple defensive layers. First, the mechanical barrier consists of a single layer of intestinal epithelial cells interconnected by tight junction proteins (including occludin, various members of the claudin family, and zonula occludens-1 (ZO-1)), which effectively restrict the paracellular passage of bacteria and endotoxins. Second, the mucus layer secreted by goblet cells acts as a chemical barrier that traps and neutralizes pathogenic microorganisms. Third, the intestinal immune compartment, which comprises structures such as Peyer’s patches and a diverse array of dendritic cells, macrophages, and T/B lymphocytes, as an immunological barrier that continuously monitors for and eliminates invading pathogens (45–50). Moreover, the normal gut microbiota, dominated by beneficial genera such as *Bifidobacterium*, *Lactobacillus*, and *Bacteroides*, not only directly reinforces these barriers but also indirectly modulates local and systemic immune responses through the production of short-chain fatty acids (SCFAs) and the regulation of bile acid metabolism (51–53). Preservation of these barrier components should be prioritized in at-risk patients to reduce invasive liver abscess incidence.

#### 3.1.2 Role of microbial metabolites in barrier maintenance and immune regulation

The fermentation of dietary fibers by the gut microbiota produces essential metabolic byproducts, primarily SCFAs such as butyrate, propionate, and acetate, that play crucial roles in multiple physiological processes. SCFAs fuel epithelial cells, enhance tight-junction protein expression, and modulate immunity via GPR41/43 activation (43, 51, 54).Under normal conditions, SCFAs regulate immune cell functions by activating G protein-coupled receptors (for example, GPR41 and GPR43). This receptor-mediated signaling cascade suppresses inflammatory responses and promotes the differentiation of T regulatory cells, ultimately maintaining a balanced local immune environment. Concurrently, the gut microbiota is pivotal in bile acid metabolism. In addition to facilitating lipid digestion and absorption, bile acids act as key signaling molecules that activate receptors such as the Farnesoid X receptor (FXR) and the G protein-coupled receptor TGR5,both of which are integral to the regulation of energy metabolism and immune modulation (55, 56).In a healthy state, a dynamic equilibrium in bile acid metabolism helps safeguard the barrier of the intestinal epithelium and regulate inflammation. Conversely, disturbances in the gut microbial ecology result in a marked reduction of SCFA production and perturbations in the composition and concentration of bile acids. These alterations directly compromise gut barrier and indirectly precipitate heightened local and systemic inflammatory responses, thereby adversely affecting the host’s immune milieu and hepatic metabolic processes. However, emerging studies report dose- and context-dependent pro-inflammatory effects of SCFAs. For example, butyrate concentrations above 5 mM activate macrophage NLRP3 inflammasomes and elevate IL-1β release (57), while acetate and propionate, though anti-inflammatory via GPR43 under homeostasis, can exacerbate Th1/Th17 responses in dysbiotic colitis models (58). Emerging data reveal context-dependent actions of SCFAs. While millimolar butyrate often promotes Treg differentiation via HDAC inhibition and enhances IL-10, concentrations above 5 mM can activate macrophage NLRP3 inflammasome and elevate IL-1β release, aggravating inflammation. Similarly, acetate and propionate via GPR43 suppress allergic Th2 responses but under dysbiotic conditions can amplify Th1/Th17 axes in colitis models. These discordant findings likely reflect differences in local SCFA concentrations, receptor expression, and the inflammatory milieu, underscoring the need for more nuanced appraisal of SCFA dosage, cell targets, and site-specific effects; critically, references 57–58 are *in vitro*/animal-based (moderate GRADE quality), with conflicting results possibly due to non-physiological doses, whereas clinical translation remains speculative without human trials. Practically, this supports dose-optimized trials of butyrate-enhancing diets or FXR agonists to restore mucosal immunity in ILA patients, potentially reducing recurrence by 20-30% based on analogous NAFLD studies. Clinically, this rationale supports trials of butyrate-enhancing diets or FXR agonists to restore mucosal immunity in ILA patients.

#### 3.1.3 Microbial dysbiosis and regulation of pathogen virulence

Under physiological conditions, the commensal microbiota functions as an effective “protective shield” by preserving the intestinal barrier and modulating local immune responses. This barrier prevents pathogens and their metabolic products from translocating across the epithelium. However, prolonged antibiotic exposure, unhealthy diets, or chronic diseases drive dysbiosis and barrier dysfunction (see Section 3.1.2), facilitating pathogen and lipopolysaccharide (LPS) translocation via the portal vein and triggering hepatic inflammation.

In summary, a balanced gut microbiota maintains effective segregation between the intestinal lumen and the internal environment through multiple barrier mechanisms and metabolic regulation, playing a pivotal role in immune homeostasis and metabolic control. Clinically, this underscores the value of therapies aimed at restoring epithelial integrity, such as FXR agonists or butyrate supplementation, to prevent bacterial translocation and mitigate ILA progression. This dysbiosis-driven barrier breakdown permits microbial products, including LPS, to reach the liver (see Section 3.2 for the ensuing inflammatory signaling cascade). Collectively, these mechanisms not only underscore the critical role of the gut–liver axis in maintaining host health but also provide a theoretical foundation for the development of precision therapeutic strategies aimed at modulating the gut microbiota, restoring barrier, and rebalancing immune responses. These insights bolster early microbiome-modulating approaches, such as probiotics or fecal microbiota transplantation (FMT), to curb hypervirulent strain overgrowth.

#### 3.1.4 Disruption of the intestinal barrier

The balance of the gut microbiota is essential for maintaining host health, yet various external and intrinsic factors can disturb this ecosystem, leading to dysbiosis, and disrupt intestinal homeostasis. First, the prolonged use of broad‐spectrum antibiotics is a major extrinsic trigger of microbial dysbiosis. Antibiotics not only deplete beneficial microbes such as *Bifidobacterium* and *Lactobacillus* but also promote the emergence of resistant strains and facilitate the spread of pathogens like *Clostridium difficile (C. difficile)*, often accompanied by drug-related toxicity (59). Second, unhealthy dietary habits significantly impair the gut ecosystem. Diets high in fat and sugar yet low in fiber, along with excessive gluten intake and vitamin D deficiency, can alter both the expression and structure of tight junction proteins and the mucus layer in epithelial cells. This disruption induces or exacerbates barrier dysfunction, ultimately leading to a decline in beneficial bacteria while allowing pathogenic organisms to proliferate (60). Moreover, chronic conditions (e.g., diabetes, obesity, and immunodeficiency) and prolonged psychological and environmental stress further compromise the stability of the intestinal microbiome (61).

Dysbiosis, characterized by loss of beneficial taxa and overgrowth of opportunistic pathogens, further impairs barrier integrity and elevates TNF-α, IL-1β, and IL-6 (see Section 3.1.2). Laboratory studies have demonstrated that under conditions of inflammation or oxidative stress, key intracellular signaling pathways (such as MAPK and NF-κB) become activated, which in turn suppresses the expression of tight junction proteins (e.g., occludin and claudin), increases intercellular gaps, and compromises the continuity of the epithelial layer (62). Simultaneously, impaired goblet cell secretion leads to a thinning of the protective mucus layer, thereby diminishing its capacity to capture and neutralize invading pathogens; persistent pro-inflammatory cytokine stimulation further induces premature epithelial cell apoptosis, exacerbating barrier breakdown (63, 64).

Collectively, these pathological changes severely compromise intestinal barrier, permitting bacterial and metabolite translocation (such as LPS). Research indicates that under dysbiotic conditions, a weakened intestinal mucosal barrier permits large quantities of bacteria and toxins to cross the epithelium into the portal circulation, thereby establishing a robust foundation for subsequent inflammatory responses and hepatic infections (26, 33). This imbalance, driven by both external insults and intrinsic pathological states, not only reduces the production of anti-inflammatory metabolites but also directly undermines barrier through the downregulation of tight junction protein expression and the thinning of the mucus layer. The resulting cascade of inflammatory reactions and immune dysregulation serves as a critical pathological link in the development of various systemic diseases, particularly invasive liver abscess and other hepatic disorders (65). Collectively, these mechanisms provide both the physical and biochemical basis for bacterial translocation, which then activates inflammatory signaling pathways within the liver. The following section will elaborate on the specific roles of these inflammatory pathways in the pathogenesis of ILA. Having established how intestinal barrier breakdown permits translocation of microbial products, we now examine the hepatic inflammatory cascades they trigger.

### 3.2 Activation of inflammatory signaling pathways

When the intestinal barrier is compromised, bacteria and their products access the portal circulation via three principal routes (16, 66): (1) Paracellular Permeation: Reduced expression of tight junction proteins and widened intercellular gaps allow bacteria and macromolecules to directly traverse the damaged epithelial layer. (2) Transcellular Transport: Certain bacteria trigger endocytic uptake and are subsequently transported across epithelial cells into the underlying lamina propria before reaching the vasculature. (3) Immune Cell-Mediated Translocation: Dendritic cells, while sampling luminal contents, internalize bacteria and then migrate to lymph nodes, effectively conveying these pathogens into the systemic circulation. Collectively, these mechanisms result in a continuous influx of bacteria and toxins, for example, LPS, into the liver, where they provoke localized inflammatory responses and infections.

During bacterial translocation, host–pathogen signaling pathways engage several key processes: (1) Cytokine and Receptor Pathways: After bacterial migration, Kupffer cells and other resident immune cells detect pathogen-associated molecular patterns (PAMPs) through toll-like receptors (TLRs), thereby rapidly triggering the NF-κB pathway. This activation leads to the robust release of proinflammatory cytokines, which not only inflict direct tissue damage but also further compromise the intestinal barrier (67, 68). (2) *In vitro* studies demonstrate that Kupffer cell–derived cytokines (TNF-α, IL-1β) triggered by LPS can increase HvKP *rmpA* and *iucA* transcription 2–4-fold, enhancing capsule thickness and biofilm biomass (69, 70). Note that these results derive exclusively from *in vitro* assays using cultured HvKP strains; definitive evidence for cytokine sensing by bacteria and subsequent virulence‐gene induction in animal models of ILA is still lacking. Whether these host cytokines directly bind bacterial two-component sensors to switch on quorum-sensing circuits *in vivo* remains to be validated. The downstream effects on adaptive immunity (Th17/Treg balance) are discussed in Section 3.3.2. This immune imbalance amplifies inflammation and pathogen virulence (see Section 3.3 for the bacteria–inflammation–virulence cycle). Based on the *in vitro* link between NF-κB–driven cytokines and HvKP virulence gene upregulation, we propose that TLR4 or NF-κB inhibitors could reduce HvKP invasiveness *in vivo*. This must be validated in animal models of ILA, measuring abscess size, bacterial load, and capsule gene expression with/without NF-κB blockade. The proinflammatory cytokine milieu thus generated (TNF-α, IL-1β, IL-6) also reshapes hepatic T-cell subsets, favoring Th17 over Treg differentiation, which we analyze in Section 3.3.2.

Collectively, these mechanisms form a self-reinforcing network that ensures the rapid and widespread activation of both local and systemic inflammatory responses following bacterial translocation. Under sustained inflammatory stress, pathogen virulence genes remain continuously upregulated, ultimately facilitating bacterial dissemination within the host and worsening clinical outcomes. From a therapeutic standpoint, targeting NF-κB or TLR4 activation could interrupt this cycle, providing a rationale for adjunctive anti-inflammatory strategies in ILA management.

**Figure 1:**
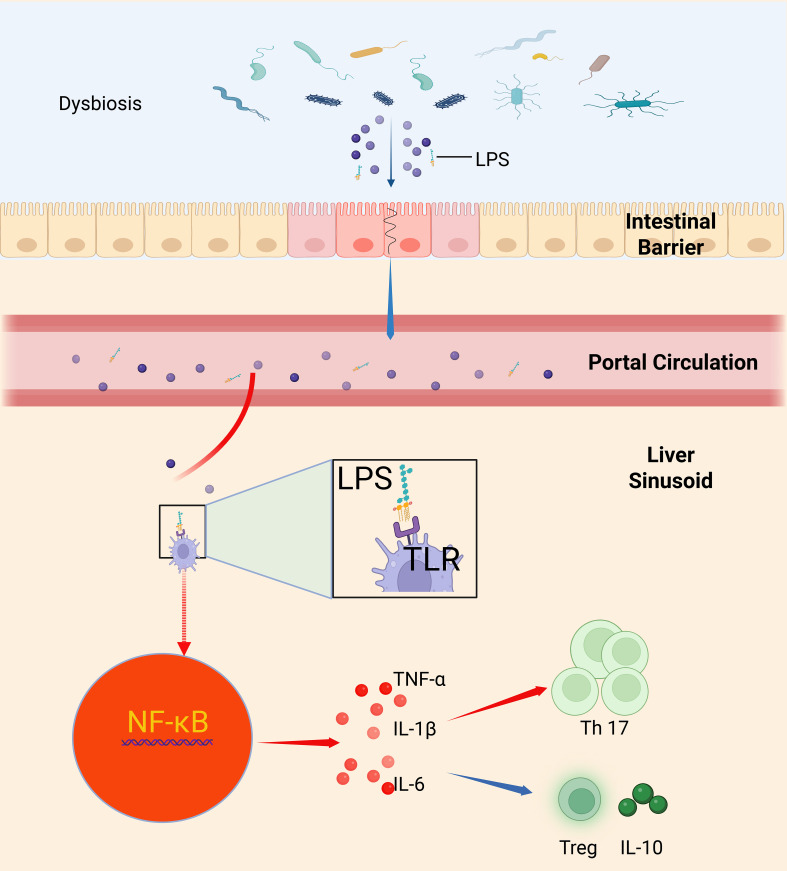
Intestinal barrier disruption and bacterial–inflammatory–immune dysregulation mediated by Kupffer cell TLR/NF-κB signaling. This schematic summarizes how intestinal barrier loss—due to disruption of tight-junction proteins (occludin, claudins, ZO-1)—allows luminal LPS to enter the portal circulation and reach the liver, where it binds Kupffer cell TLRs to trigger NF-κB activation and secretion of TNF-α, IL-1β, and IL-6; this proinflammatory milieu expands Th17 cells while depleting regulatory T cells and IL-10, tipping hepatic immunity toward inflammation, upregulating pathogen virulence genes, and driving invasive liver abscess formation (35, 71, 113).

After disruption of the intestinal barrier, microbial components and toxins from the gut gain access to the liver, where they interact with resident immune cells such as Kupffer cells. LPS, a prototypical endotoxin, activates host TLRs, primarily triggering a downstream NF-κB signaling cascade and resulting in the robust secretion of proinflammatory cytokines including TNF-α, IL-1β, and IL-6. This proinflammatory milieu is typically accompanied by an expansion of Th17 cells, while the population of Treg cells is relatively diminished, thereby disturbing immune homeostasis. The proliferation of Th17 cells is closely associated with increased levels of IL-17; IL-17 not only exacerbates local inflammation but also induces the secretion of additional inflammatory mediators, further promoting the upregulation of pathogen virulence genes. In contrast, Treg cells help suppress excessive inflammation through the secretion of anti-inflammatory cytokines such as IL-10; a decline in their numbers impairs the effective control of the inflammatory response (35, 71) (**Figure 1**).

### 3.3 The bacteria–inflammation–virulence cycle

Based on robust *in vitro* evidence but limited *in vivo* data, we propose the following feedback loop: Barrier failure and inflammation establish the bacteria–inflammation–virulence cycle. Dysbiosis not only impairs barrier, facilitating the translocation of harmful microbes and their metabolites into the portal circulation, but also triggers the host to produce large amounts of proinflammatory mediators (72). We hypothesize that this cytokine‐driven cycle upregulates HvKP virulence genes *in vivo* and amplifies bacterial invasiveness; direct validation in ILA animal models remains an urgent priority.

#### 3.3.1 Regulation of bacterial virulence gene expression in invasive liver abscess

**Figure 2:**
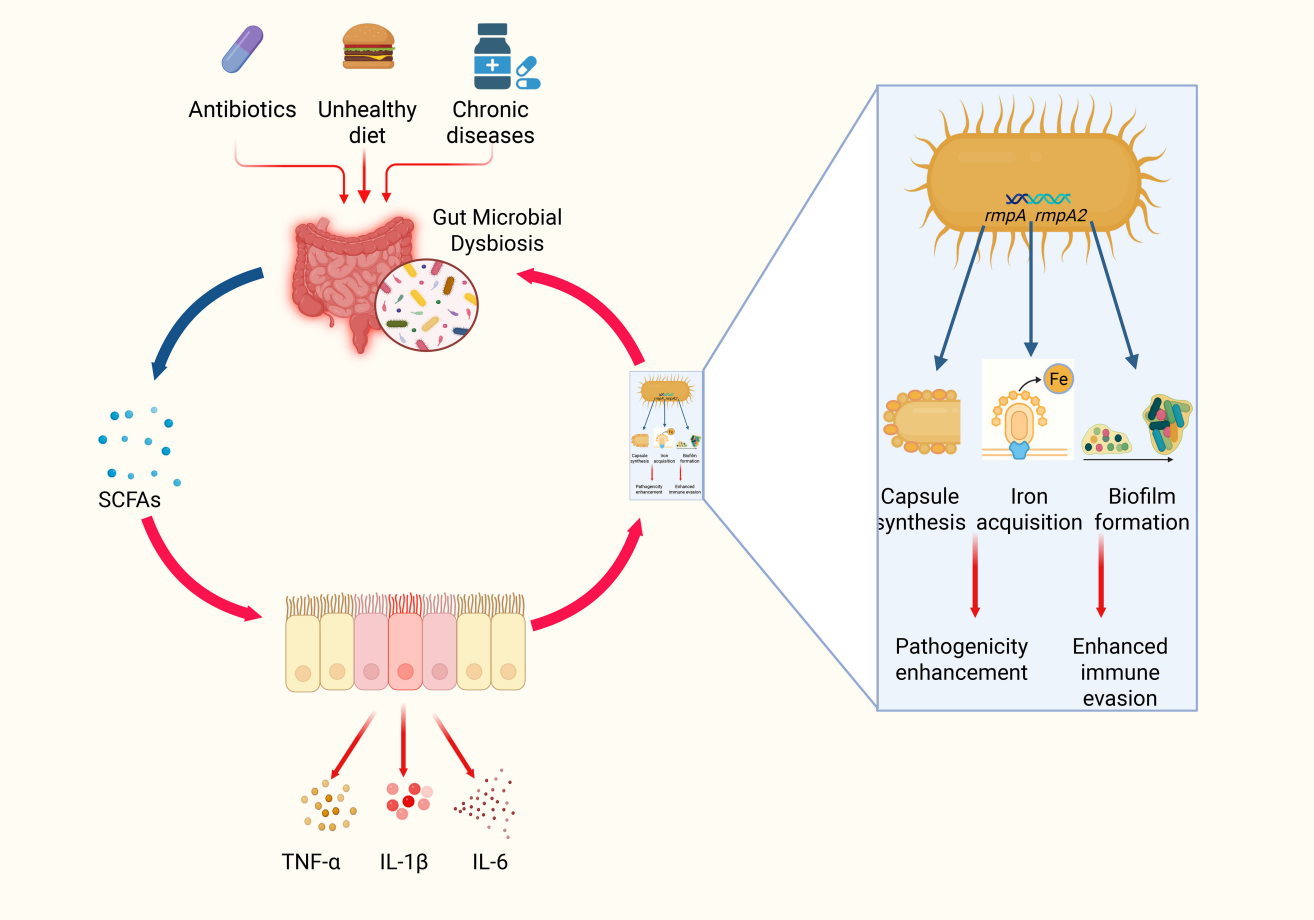
Schematic diagram of gut microbial dysbiosis and the bacteria–inflammation–virulence cycle of HvKP. In this three‐step schematic, a healthy gut, rich in *Bifidobacterium* and *Lactobacillus*, produces abundant SCFAs that maintain tight‐junction integrity and block bacterial translocation; prolonged antibiotic use, poor diet, and chronic disease then induce microbial dysbiosis, sharply reducing SCFAs and triggering TNF-α, IL-1β, and IL-6 release to create a proinflammatory microenvironment; finally, these inflammatory cues activate hypervirulent *Klebsiella pneumoniae* to upregulate *rmpA*, *rmpA2*, and *iucA*, driving capsule overproduction and biofilm formation that enhance immune evasion and facilitate systemic dissemination (69, 114, 115).

In a dysbiotic milieu, the loss of anti-inflammatory mediators exacerbates both intestinal barrier damage and local inflammation (see Section 3.1.2). As a result, the inflammatory state is accompanied by elevated secretion of TNF-α, IL-1β, and IL-6. These cytokines, in turn, trigger upregulation of bacterial virulence genes. Preclinical models show that HvKP cultured with exogenous TNF-α or IL-6 upregulates capsule regulator *rmpA* by 3-fold and siderophore gene *iucA* by 2.5-fold, as measured by qRT-PCR and capsule staining (73). It is not yet known which bacterial receptor(s) sense these cytokines or how this occurs in the infected liver microenvironment. These ‘preclinical models’ refer exclusively to *in vitro* cultures; analogous experiments in murine or other ILA models have not yet been reported, leaving a critical gap in translational relevance. Moreover, HvKP exhibits pronounced pathogenicity; in the presence of inflammatory mediators, its virulence genes (such as *rmpA*, *rmpA2*, and *iucA*) are significantly upregulated. This upregulation not only augments capsular synthesis but also reinforces the protective properties of biofilms, thereby improving bacterial survival and facilitating their spread within host tissues (74) (**Figure 2**).

#### 3.3.2 Interactive regulation of inflammatory and immune signaling in invasive liver abscess

Building on the cytokine milieu described in Section 3.2, elevated TNF-α, IL-1β, and IL-6 bias CD4_+_ T-cell differentiation toward Th17 at the expense of Treg, further amplifying local inflammation. *In vitro* assays on HvKP K1/ST23 strains demonstrate that IL-17 supplementation at 10–50 ng/mL increases *rmpA* promoter activity by ~50% via AI-2 quorum signals (75). Whether IL-17 similarly regulates *iucA* expression or functions across diverse HvKP lineages *in vivo* has not yet been established. This Th17/Treg imbalance constitutes the immune arm of the bacteria–inflammation–virulence feedback loop.

Current evidence indicates that in invasive liver abscess, gut dysbiosis combined with local inflammatory conditions, mediated by host factors, significantly upregulates the expression of pathogen virulence genes. This process not only augments pathogen invasiveness but also reinforces the bacteria–inflammation–virulence cycle described in Section 3.3, thereby driving further disease progression. Preclinical models have demonstrated that disrupting the ‘dysbiosis–virulence upregulation–inflammation’ loop using anti-inflammatory agents or quorum-quenching compounds, can attenuate HvKP pathogenicity. Armed with these molecular and immunological insights into the bacteria–inflammation–virulence cycle, we can now explore how they inform novel and multi‐targeted treatment strategies.

## 4 Treatment strategies

Currently, clinical management of invasive liver abscess largely relies on conventional antimicrobial therapy and interventional drainage procedures. However, as our understanding of the gut–liver axis and microbial dysbiosis in the disease’s pathogenesis deepens, the limitations of traditional approaches have become increasingly evident. Although standard antibiotic regimens can effectively suppress pathogen proliferation in the short term, their efficacy is compromised by the persistent emergence of resistant strains, inadequate penetration of drugs into localized lesions, and suboptimal modulation of host immune responses (76). Simultaneously, while interventional treatments, including surgical drainage and percutaneous techniques, can relieve abscess pressure and clear local infections, they are associated with high procedural risks, significant trauma, and elevated recurrence rates. Moreover, these methods seldom address the foundational issues of dysbiosis and disruption of the gut–liver axis functionality (77). Recent multi-level network analyses have demonstrated that in patients with invasive liver abscess, both an imbalance in the gut microbiota and compromised intestinal barrier not only facilitate the translocation of pathogens via the portal vein to the liver, thus triggering local infection, but also activate immune and inflammatory responses through the gut–liver regulatory system, further exacerbating pathological damage (37, 78, 79). Consequently, there is a pressing need for innovative treatment strategies that control the infection while simultaneously restoring the dynamic equilibrium of the intestinal microbiota.

### 4.1 Limitations of conventional antimicrobial and interventional therapies

Conventional antibiotic therapy suffers from several significant limitations. Firstly, the emergence of drug resistance remains a major challenge (80, 81). Prolonged or excessive use of broad-spectrum antibiotics can promote the selective growth of resistant strains, thereby diminishing the drugs’ effectiveness; however, these studies (80, 81) are retrospective meta-analyses (moderate GRADE quality), potentially biased by reporting inconsistencies, limiting firm conclusions on resistance rates. Secondly, inadequate drug penetration poses a further obstacle (82); antimicrobials often fail to adequately infiltrate abscess cavities due to poor local blood supply, the unique microenvironment within the abscess, and complex microbial interactions, making it difficult to reach optimal bactericidal concentrations. Thirdly, antibiotic monotherapy does not sufficiently modulate the immune response, leaving underlying dysbiosis and intestinal barrier damage, critical factors in gut–liver axis dysfunction, largely unaddressed (83, 84); clinically, this suggests transitioning to combination therapies post-initial control, though evidence is low-quality observational.

Similarly, interventional treatments such as surgical or percutaneous drainage (85–87), although effective in rapidly reducing abscess pressure and alleviating local inflammation, are burdened by drawbacks. These procedures are associated with considerable trauma, a high risk of recurrence, and the potential for secondary activation of the immune system due to the release of inflammatory mediators. Consequently, relying solely on these methods does not fundamentally restore the balance of the gut–liver axis nor address the inherent link between bacterial translocation and abscess formation (84). Notably, emerging gut-modulatory interventions like FMT still lack long-term safety and efficacy data outside of rCDI, underscoring the need for rigorously designed, registry-based clinical trials before wider adoption. This gap highlights the need for combined approaches antibiotics plus gut-modulatory therapies, to both clear infection and recalibrate the host immune–microbiome interface, with practical applications like stepwise protocols: antibiotics first, then FMT for dysbiosis. Given these therapeutic gaps, recent efforts have shifted toward microbiome-modulating and multi-target approaches, as detailed below.

### 4.2 Exploration of novel therapeutic strategies

In view of the limitations of conventional treatments, recent research has increasingly focused on innovative strategies that modulate the gut microbiome, enhance intestinal barrier, and employ multi-target combination interventions. The central concept of these approaches is to achieve synergistic therapeutic effects through the integration of internal and external mechanisms.

#### 4.2.1 Probiotics treatment

Probiotics, as live microorganisms, can favorably alter the composition of the gut microbiota, boost the production of SCFAs, modulate bile acid metabolism, and attenuate local inflammatory responses. Collectively, these actions contribute to restoring barrier and indirectly impeding the translocation of pathogens. The specific mechanisms include (88–91): (1) Optimizing Microbial Structure: Probiotics increase the proportion of beneficial bacteria while suppressing the growth of opportunistic pathogens, thereby re-establishing microbial equilibrium. (2) Strengthening the Intestinal Barrier: By promoting mucosal repair and upregulating the expression of tight junction proteins, probiotics reduce intestinal permeability, limiting the passage of pathogens and endotoxins into the portal venous system. (3) Immune Regulation: Probiotics activate both local and systemic immune responses by balancing the secretion of pro- and anti-inflammatory cytokines, which not only diminishes local inflammation but also enhances overall antimicrobial defense. Early probiotic administration, when combined with antibiotics, may reduce ILA recurrence (34, 35).

#### 4.2.2 FMT

FMT involves transferring a complete, healthy microbial community from a donor into the recipient’s gut to rapidly re-establish the native ecosystem. Its primary advantages include (92–96): (1) Rapid Restoration of Microbial Diversity: FMT swiftly corrects dysbiotic conditions by reconstituting the recipient’s gut microbiota, thereby enhancing intestinal barrier. (2) Regulation of the Gut–Liver Axis: By improving gut ecology and restoring barrier, FMT reduces the risk of endotoxin and harmful metabolite translocation through the portal vein, ultimately mitigating hepatic inflammation and fibrosis. (3) Personalized Treatment Potential: With careful donor screening and individualized analysis, FMT provides a promising platform for precision medicine. Nevertheless, results across indications are heterogeneous. Meta-analyses in irritable bowel syndrome report symptom remission rates from 0 to 50% with overall low–moderate GRADE confidence, largely driven by small RCTs, variable donor screening, and inconsistent administration routes (97).

Meta-analyses of FMT in recurrent *C. difficile* infection report cure rates above 80% (98), but often note only moderate to low GRADE confidence due to small sample sizes, open-label designs, and heterogeneous endpoints. In non-rCDI indications, ulcerative colitis, irritable bowel syndrome, and metabolic syndrome, randomized, placebo-controlled trials yield mixed outcomes (25–30% remission vs. null effects) (99). Most adverse events are mild gastrointestinal symptoms, yet case reports document serious infections (bacteremia, viral transmission) and several FMT-associated deaths (100). Off-target engraftment (“terraforming”) in non-colonic sites may provoke persistent metabolic or immunologic shifts. Accordingly, FMT for conditions beyond rCDI should remain investigational, with standardized donor screening, rigorous blinded RCTs including long-term follow-up, and centralized adverse-event registries. Moreover, off-label FMT use has been linked to serious adverse events, including bacteremia and fatal infections due to insufficient donor screening, and persistent off-target engraftment causing metabolic or immunologic shifts. The absence of centralized safety registries magnifies these concerns.

#### 4.2.3 Multi-target combination therapy

Modern therapies emphasize comprehensive (101, 102), multi-level interventions for disease control. Multi-target therapy pairs conventional antibiotics and drainage with probiotics or FMT.

This dual approach delivers both rapid pathogen control and long-term microbiome restoration. Synergistic effects are achieved through: (1) Dual Action on Infection and Microbial Regulation: Early administration of antibiotics alongside interventional techniques effectively reduces pathogen loads, while subsequent use of probiotics or FMT reconstructs the microbial community for long-term stability. (2) Reduction in Resistance Risk: By allowing each treatment modality to operate at lower doses in a synergistic manner, multi-target strategies help decrease the emergence of drug resistance typically associated with long-term monotherapy (93, 103). (3) Comprehensive Restoration of the Gut–Liver Axis: Systematic treatment enhances intestinal barrier, modulates local immune responses, and re-establishes overall microbial equilibrium, thereby offering sustained, holistic protection for the patient (84, 104).

Integrating mechanistic and clinical insights via multi-omics and systems modeling paves the way for precision, multi-target interventions. To facilitate comparison and highlight the intrinsic connections and synergistic regulatory mechanisms among these strategies, the table below summarizes the key characteristics, mechanisms of action, and limitations of each treatment modality (**Table 4**).

**Table 4:** Treatment strategies and their characteristics.

Treatment strategy	Intervention mechanism and principal effects	Synergistic outcomes and advantages	Limitations and disadvantages
Traditional Antibiotic Therapy	Uses antimicrobial agents to directly eliminate pathogens from the affected tissues.	Enhances pathogen clearance when complemented by the host’s immune response.	Associated with antibiotic resistance and potential adverse effects (105).
Interventional Procedures (106)	Employs minimally invasive techniques (e.g., drainage or mechanical removal) to eliminate infectious foci and reduce pathogen burden.	Provides rapid relief from localized infections, especially where antibiotics alone may be insufficient.	Involves procedural risks and may lead to complications due to invasive nature.
Probiotic Interventions (8)	Introduces beneficial microorganisms to rebalance the gut microbiota and reinforce the mucosal barrier.	Can work synergistically with the host’s natural immune mechanisms and enhance gut-liver interactions.	Effectiveness varies, being highly, dependent on the specific strains used and individual patient factors.
FMT (93)	Restores a balanced intestinal microbiome by transferring a donor’s microbial community, thereby normalizing metabolic and immunologic functions.	Complements conventional therapies and helps reduce infection recurrence through a more holistic approach.	Faces regulatory challenges and its long-term safety profile remains to be fully established (107).
Comprehensive Multi-Strategy Approach	Integrates antimicrobial, interventional, and microbial modulation strategies into a unified, patient-tailored treatment regimen.	Leverages the combined merits of diverse modalities to optimize therapeutic outcomes.	Increases implementation complexity and costs, with a higher potential for interactions between drugs or treatment techniques.

### 4.3 Integration of mechanisms and future perspectives

As illustrated in the table above, each therapeutic strategy for managing invasive liver abscesses utilizes a distinct mechanism of action with its own advantages and inherent limitations. For example, conventional methods offer rapid infection control; however, they do not fundamentally modulate the gut–liver axis or restore microbial balance. In contrast, probiotic therapy and FMT aim to reestablish the endogenous microecology by reversing pathological conditions through biological regulation. Meanwhile, multi-target combination therapies seek to integrate the strengths of both approaches, delivering timely and precisely dosed interventions that achieve comprehensive control with minimal therapeutic input.

Furthermore, the clinical application of these novel strategies requires overcoming the challenges inherent in integrating diverse interventional modalities. For example, determining how best to combine antimicrobial and interventional techniques during the acute phase with the timely initiation of probiotic or FMT treatments, and establishing optimal transition timings and dosing standards, will necessitate support from multi-center, large-sample clinical trials (108). In parallel, advancements in artificial intelligence and multi-omics technologies are paving the way for the development of multi-layered intervention models via computer simulation and network pharmacology. Such progress is expected to provide both the theoretical foundation and technical support needed to design individualized, multi-target combination therapies (109–112). A comparative analysis of traditional antimicrobial and interventional approaches versus probiotic, FMT, and multi-target strategies reveals that while each method offers specific benefits, single modalities often fail to concurrently address both infection and microbial dysbiosis. To advance precision microbiome therapies with FMT and SCFA interventions, future studies should prioritize: 1) establishing multicenter FMT registries that track long-term outcomes, including infectious complications and metabolic sequelae, to comprehensively assess safety and efficacy; 2) performing SCFA dose–response mapping in humanized gut-immune co-culture models to delineate pro- versus anti-inflammatory thresholds; 3) developing and standardizing *in vivo* SCFA quantitation protocols alongside robust profiling of GPR41/43 receptor expression and signaling in target tissues; and 4) integrating AI-driven multi-omics analyses to predict individual host responses to FMT and SCFA treatments, thereby laying the groundwork for truly personalized microbiome-based medicine.

## 5 Discussion and future perspectives

In conclusion, this synthesis of the literature indicates our multi-layer analysis shows that gut–liver axis disruption drives invasive liver abscess. The impairment of barrier and the ensuing microbial dysbiosis facilitate the translocation of harmful bacteria and their toxins into the liver. This event initiates a cascade of inflammatory responses through the activation of hepatic immune cells, which in turn upregulates key bacterial virulence factors. Such a pathological cascade not only intensifies liver tissue injury but also promotes rapid and systemic dissemination of the infection; critically, while mechanistic evidence (*in vitro*/animal) firmly supports this cascade, clinical data is graded low-moderate under GRADE due to observational biases, highlighting speculation in human applicability.

Future studies should explore deeper signaling networks in host–pathogen interactions to identify key factors in virulence regulation, barrier repair, and immune modulation. These insights could guide early diagnosis and personalized interventions for ILA, such as biomarker-based screening for dysbiosis in diabetic patients. Bridging these divergent findings will require coordinated clinical trial frameworks. For FMT, future studies must standardize donor selection criteria, delivery methods, and efficacy endpoints. In SCFA research, dose-response mapping across physiological (0.5–5 mM) and pharmacological (>5 mM) concentrations in humanized gut models is essential. Moreover, large-scale registries with uniform adverse-event reporting and integrated biomarker panels are needed to delineate context-dependent roles of SCFAs and optimize microbiome-based interventions. In summary, this review elucidates the pathological significance of gut–liver axis dysregulation in ILA and reveals complex disturbances in signaling and inflammation driven by microbial imbalance. It further discusses emerging therapeutic strategies, such as probiotics, FMT, and multi-target combination therapies, that hold promise for improving patient outcomes and reducing the risk of recurrence, with practical applications like integrated protocols reducing mortality by targeting both infection and dysbiosis. Looking forward, addressing challenges in sample and data standardization as well as cross-scale integration will be critical for building more precise and comprehensive models of ILA pathogenesis, thereby laying a solid theoretical foundation for individualized precision therapies. Bridging these mechanistic insights with coordinated clinical trials and biomarker-driven endpoints will be crucial to translate our findings into patient benefit.
